# Proteomics promises a new era of precision cancer medicine

**DOI:** 10.1038/s41392-019-0046-9

**Published:** 2019-05-03

**Authors:** Chong Wu, Limin Zheng

**Affiliations:** MOE Key Laboratory of Gene Function and Regulation, State Key Laboratory of Biocontrol School of Life Sciences, Sun Yat-sen University, 510275 Guangzhou, China

**Keywords:** Molecular medicine, Systems biology, Gastrointestinal cancer

A study recently published in *Nature* provides new insights into the proteomic heterogeneity of early-stage hepatocellular carcinoma related to hepatitis B virus infection.^1^ Based on quantitative proteomic and phospho-proteomic profiling, early-stage hepatocellular carcinoma can be stratified into subtypes with different clinical outcomes, and a druggable target has been identified.

Hepatocellular carcinoma (HCC) is one of the leading causes of tumor-related death worldwide and mostly results from viral infection and liver cirrhosis.^2^ The highest incidence rates of HCC are in Asia and sub-Saharan Africa, on account of the high prevalence of hepatitis B virus (HBV) infection.^2^ In the past decades, markedly increased numbers of HCC cases were diagnosed at earlier stages, owing to the improved surveillance and advances in imaging technologies. However, the treatment options for early-stage HCC are still limited. Due to the shortage of liver donors, surgical resection remains the primary treatment for early-stage HCC. Although early-stage HCC patients have a relatively favorable prognosis in general, about half of them suffer from rapid postoperative recurrence, resulting in a 5-year survival rate of less than 30% in this subset of patients.^3,4^ Therefore, it is clinically important to identify the molecular subclasses of these heterogeneous HCCs as well as potential novel therapeutic targets for adjuvant therapies.

**Fig. 1:**
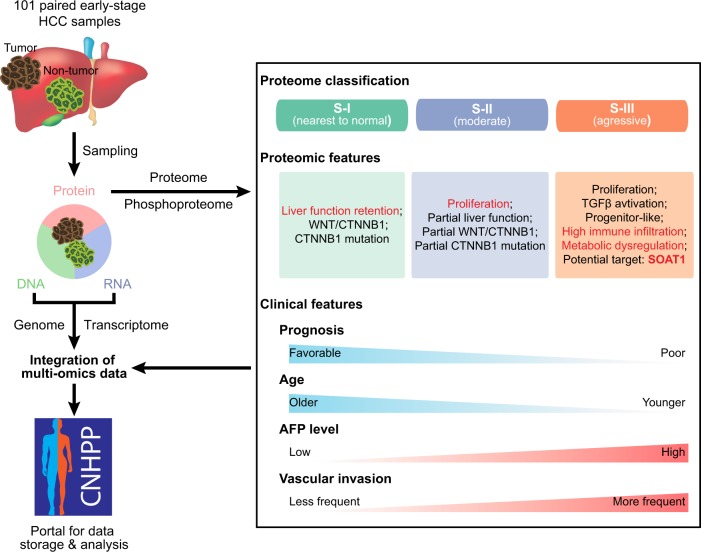
Overview of multi-omics analyses of HBV-related early-stage HCC. The proteomic and phospho-proteomic landscapes of 101 paired tumor and nontumor tissues of early-stage HCC were examined. Based on these data, early-stage HCCs were stratified into three major proteomic subtypes: S-I, S-II, and S-III. All three subtypes of HCC were characterized, and the drug-targetable candidate protein SOAT1 was identified in S-III. They also integrated the proteome information with genome and transcriptome data, and performed a comparative analysis, mapping the new-dimensional findings onto the literature-derived classifications. All the multi-omics data are stored in a data portal at http://liver.cnhpp.ncpsb.org/

A study recently published in *Nature* sheds new light on the proteomic subtypes of HCC related to HBV infection Fig. 1. In this study, Jiang et al.^1^ characterized the patterns of signatures and pathways that are altered between different proteomic subtypes of early-stage HCC using quantitative proteomic and phospho-proteomic analyses. In total, 101 paired tumor and nontumor tissues of early-stage HCC related to hepatitis B virus infection were examined. Interestingly, proteomic analysis revealed that tumors, in particular those showing more aggressive features, expressed a significantly higher number of proteins than did the paired nontumor tissues. The phospho-proteome profiling additionally showed the hyperphosphorylation of signaling pathways, including those involved in inflammation and cell metastasis, in tumor regions.

To decipher the heterogeneity of early-stage HCC tumors, Jiang et al.^1^ used a nonnegative matrix factorization consensus-clustering analysis to stratify the tumor cohort into three major proteomic subtypes, namely, subtypes S-I, S-II, and S-III. HCC of subtype S-III has the worst prognosis, with more aggressive characteristics than either the S-I or S-II subtypes. These characteristics include the upregulation of proteins with unfavorable prognostic influence (TGFβ1, KRT19, and MMP9), activation of pathways associated with disease progression (HIF1, integrin and Rho GTPases pathways), and enrichment of established transcriptomic signatures of aggressive HCC subclasses that were specifically found in the S-III subtype.

Given that the worst postsurgical prognoses were found for subtype S-III, it is reasonable to propose adjuvant therapies for this subtype. To this end, a cholesterol acyltransferase—sterol O-acyltransferase 1 (SOAT1)—was identified as a potential target. The upregulation of SOAT1 was associated with the greatest risk of a poor prognosis after resection. Both SOAT1 knockdown and treatment with avasimibe, a SOAT1 inhibitor, reduced the cholesterol levels in the plasma membrane, inhibited the integrin and TGFβ signaling pathways, and ultimately suppressed the proliferation and migration of HCC cells. The therapeutic effectiveness of avasimibe was further validated in patient-derived tumor xenograft models, suggesting that SOAT1 may serve as a promising therapeutic target in adjuvant therapy for the most aggressive S-III HCCs.

Interestingly, S-III subtype HCC also exhibits signatures of tumor-promoting immune activities.^5,6^ and demonstrates upregulation of immune checkpoint molecules. These immune patterns are reminiscent of the predictive biomarkers for immune checkpoint blockade, which has recently shown promise with regard to HCC treatment.^7,8^ Whether and how the proteomic profiling data may facilitate the development of precision strategies and/or predictive biomarkers for immunotherapies remains an important area of future studies with great potential.

For decades, systems biology has been driven primarily by genomic technologies, at least partly due to a lack of methods for measuring actual effector molecules, i.e., proteins and metabolites,^9^ with comparable depth and throughput. Today, owing to the development of mass spectrometry, obtaining rapid and relatively deep proteome characterization is within reach. This study not only exemplifies how high-throughput proteomic profiling is poised to deliver novel insights into tumor heterogeneity but also highlights the promise of a new era of proteomics-driven precision medicine. In this coming era, systems-level, high-resolution investigations into proteins and their posttranslational modifications will undoubtedly facilitate more precise targeted and immune cancer therapies.
